# Spontaneous NF-κB Activation by Autocrine TNFα Signaling: A Computational Analysis

**DOI:** 10.1371/journal.pone.0078887

**Published:** 2013-11-11

**Authors:** Jakub Pękalski, Pawel J. Zuk, Marek Kochańczyk, Michael Junkin, Ryan Kellogg, Savaş Tay, Tomasz Lipniacki

**Affiliations:** 1 Institute of Fundamental Technological Research, Polish Academy of Sciences, Warsaw, Poland; 2 Institute of Physical Chemistry, Polish Academy of Sciences, Warsaw, Poland; 3 Institute of Theoretical Physics, Faculty of Physics, University of Warsaw, Warsaw, Poland; 4 Department of Biosystems Science and Engineering, ETH Zurich, Zurich, Switzerland; 5 Department of Statistics, Rice University, Houston, Texas, United States of America; Universitat Politecnica de Catalunya, Spain

## Abstract

NF-κB is a key transcription factor that regulates innate immune response. Its activity is tightly controlled by numerous feedback loops, including two negative loops mediated by NF-κB inducible inhibitors, IκBα and A20, which assure oscillatory responses, and by positive feedback loops arising due to the paracrine and autocrine regulation via TNFα, IL-1 and other cytokines. We study the NF-κB system of interlinked negative and positive feedback loops, combining bifurcation analysis of the deterministic approximation with stochastic numerical modeling. Positive feedback assures the existence of limit cycle oscillations in unstimulated wild-type cells and introduces bistability in A20-deficient cells. We demonstrated that cells of significant autocrine potential, i.e., cells characterized by high secretion of TNFα and its receptor TNFR1, may exhibit sustained cytoplasmic–nuclear NF-κB oscillations which start spontaneously due to stochastic fluctuations. In A20-deficient cells even a small TNFα expression rate qualitatively influences system kinetics, leading to long-lasting NF-κB activation in response to a short-pulsed TNFα stimulation. As a consequence, cells with impaired A20 expression or increased TNFα secretion rate are expected to have elevated NF-κB activity even in the absence of stimulation. This may lead to chronic inflammation and promote cancer due to the persistent activation of antiapoptotic genes induced by NF-κB. There is growing evidence that A20 mutations correlate with several types of lymphomas and elevated TNFα secretion is characteristic of many cancers. Interestingly, A20 loss or dysfunction also leaves the organism vulnerable to septic shock and massive apoptosis triggered by the uncontrolled TNFα secretion, which at high levels overcomes the antiapoptotic action of NF-κB. It is thus tempting to speculate that some cancers of deregulated NF-κB signaling may be prone to the pathogen-induced apoptosis.

## Introduction

### NF-κB Regulatory System

Innate immunity forms the first line of defense against pathogens. In the first phase, cells detect pathogens with their membrane and cytoplasmic receptors. This leads to the activation of transcription factors from the NF-κB, IRF and AP-1 families. These factors jointly regulate the activity of several hundred genes responsible for inflammation, antiviral protection, proliferation and apoptosis. In particular, they induce the production of pro-inflammatory cytokines like IL-1, TNFα, as well as IFN-α and IFN-ß. Secretion of these cytokines leads to the second phase of the cellular innate immune response in cells that have not yet encountered the pathogen. The cytokine-activated cells may themselves produce and secrete the same cytokines leading to the spread of paracrine signaling [1], [2] or to augmenting and stabilizing signaling in the secreting cells via autocrine regulation [3], [4]. In the current study, the focus is on the analysis of TNFα autocrine regulation in the NF-κB pathway.

NF-κB regulates numerous genes important for pathogen- or cytokine-induced inflammation, immune response, cell proliferation and survival (reviewed in [5], [6]). Nuclear activity of NF-κB is tightly controlled by negative feedback loops mediated by NF-κB-responsive proteins: IκBα [7]–[9], IκBϵ [8], [10], [11] and A20 [12]–[14]. These negative feedback loops lead to oscillatory responses, in which NF-κB circulates between the cytoplasm and nucleus with the period of about 100 min [8]. The primary inhibitors, IκBα and IκB, directly bind to NF-κB, inhibit its transcriptional activity and transport it back to the cytoplasm. Interestingly, expression of IκBϵ is delayed with respect to IκBα [11], which increases desynchronization of cells and leads to damping of oscillations observed at the population level, resulting in robust tissue responses [15]. A20 mediates the outer negative feedback loop by attenuating the catalytic activity of the IKK complex (consisting of IKKγ, also called NEMO, IKKα and IKKß). In A20-deficient cells the IKK activity remains at a high level preventing the accumulation of inhibitors IκBα and IκBϵ [14]. This leads, in turn, to the elevated NF-κB transcriptional activity and causes chronic inflammation. There are at least two levels of A20-mediated regulation of IKK complex activity: (1) A20 directly interacts with the IKK complex reducing its catalytic activity [16]–[18] and (2) A20 primes TNF receptor interacting protein (RIP) for degradation, and thus attenuates TNF receptor downstream signaling [19].

Regarding the direct regulation mode, A20 binds to IKKγ and speeds up further phosphorylation of active IKKß kinase into the inactive form [16], [20]. (IKKß activation proceeds via phosphorylation at Ser-177 and Ser-181, but further phosphorylation at the C-terminal serine cluster inhibits its catalytic activity [20].) Later, it was found that A20 and ABIN-1 bind to the IKK complex, and A20 inhibits activation of NF-κB by de-ubiquitination of IKKγ [17], reviewed recently in [21]. (Lys-63-linked ubiquitination of IKKγ is an important step for the activation of IKK and NF-κB following various stimuli, including TNFα [22].) Interestingly, A20 itself is a putative substrate of IKKß, which phosphorylates A20 on Ser-381, thereby increasing its ability to downregulate NF-κB in response to multiple stimuli [23]. Recently, Skaug et al. reported a direct non-catalytic mechanism of IKK inhibition by A20 showing that overexpressed A20 impaired IKK activation without reducing RIP1 ubiquitination [18].

Regarding the indirect IKK regulation mode, A20 acts as a ubiquitin editing protein: it removes Lys-63-linked ubiquitin chains from RIP and then functions as a ubiquitin ligase by polyubiquitinating RIP with Lys-48-linked ubiquitin chains, thereby targeting RIP for proteasomal degradation, and thus attenuating TNFR1 receptor signaling [19], reviewed in [24], [25]. The modeling studies showed distinctive roles of these two, direct and indirect, modes of regulation [26], [27]. The direct mode allows for the termination (or strong reduction) of IKK activity after A20 is synthesized (which takes about 1 hour) [26], while the second mode renders cells less sensitive to subsequent pulses of TNFα, if these pulses are separated by a short timespan [27].

Later studies showed that the role of A20 goes beyond the control of NF-κB and that A20 is a general inhibitor in innate immune signaling; it protects cells from chronic inflammation, endotoxic shock and plays a role of tumor suppressor [28], [29]. In particular, A20 inhibits IRF3/IRF7 signaling [30], [31]. Similarly as for the NF-κB pathway, it acts upstream of the TBK1–IKKϵ–IKKγ complex regulating negatively retinoic acid-inducible gene I protein (RIG-I) [32], and potentially may act at the level of this complex by binding to IKKγ [31].

As said, the negative feedback loops involving IκBα and A20 lead to oscillatory responses. These oscillations appear damped when analyzed at the population level, but single cell experiments by Nelson et al. on SK-N-AS cells and Tay et al. on 3T3 cells demonstrated that oscillations persist at least up to 10 hours [33], [34]. Discrepancy between population- and single cell-based observations can be explained by the progressing desynchronization of cells in the population [35], [36], although the controversy about reconciling single cell and population data still exists [37]. The major objection towards single cell experiments is that the additional gene copies coding for fluorescently tagged NF-κB may alter dynamics of the whole system. However, both experimental [38] and modeling studies [39] show that the number of NF-κB gene copies or its expression level influences only the amplitude but not the period of oscillations. Moreover, in our recent experiment [34], the expression of NF-κB remained practically unchanged due to the knockout of endogenous RelA, yet the oscillatory pattern was still clearly visible for 10 ng/ml TNFα dose.

### TNFα Autocrine and Paracrine Signaling

TNFα affects growth, differentiation and function of cells of many types, and is a major mediator of inflammatory immune responses [40], [41]. It is considered as a key mediator of the septic shock syndrome induced by either LPS or bacterial superantigens [42], [43]. The potent activating abilities of TNFα are transmitted by 2 distinct cell-surface receptors: TNFR1 and TNFR2; the first one binds TNFα molecules with higher affinity [44] and is considered responsible for the most of TNFα-induced signaling [45]. It is established that binding of TNFα initiates protein–protein interactions between TNFR1 and the TNFR-associated death domain protein (TRADD). TRADD in turn recruits receptor-interacting protein (RIP) and TRAF2 for NF-κB and survival signals [46], [47].

The TNFα autocrine and paracrine signaling arises since TNFα-inducible NF-κB serve itself as a primary transcription factor for TNFα. Over twenty years ago Collart et al. showed that TNFα promoter contains four κB motifs that can bind constitutive and inducible forms of NF-κB [48]. Further analysis of κB motives in TNFα promoter revealed that two sites, κB2 and κB2a, play a primary role in TNFα regulation by NF-κB in response to LPS stimulation in human monocytes [49].

The autocrine regulation was observed in various cell lines and tissues: first, Wu et al. showed that TNFα functions as autocrine and paracrine growth factor in ovarian cancer [50]. Coward et al. and Guergnon et al. demonstrated that TNFα induces TNFα synthesis via NF-κB activation in human lung mast cells and B cells [51], [52]; Nadeau and Rivest found that *in vivo* TNFα injection induced TNFα mRNA expression in microglia and astrocytes [53], and later Kuno et al. showed that the activation of microglia by LPS is partially mediated by microglia-derived TNFα, confirming the existence of a positive feedback loop [54]. Hu et al. demonstrated that autocrine TNFα signalling (via NF-κB) mediates endoplasmic reticulum stress-induced cell death [55]. Recently, Rushworth et al. reported the autocrine TNF signaling (via NF-κB) in monocytes: TNF stimulation leads to sustained production of TNF mRNA for 48 hours; the NF-κB inhibition suppresses the TNF autocrine regulation [56]. Although observed in many cell lines, strength of the autocrine and paracrine TNFα signaling is cell line-specific. Cells can be characterized by their autocrine potential based on their ability to secrete TNFα and by their sensitivity to TNFα stimulation controlled primarily by the TNFR1 level.

Autocrine TNFα signaling may start spontaneously or in response to numerous stimuli, including TNFα itself, other cytokines, or LPS. The spontaneous activation of the NF-κB signaling pathway was observed in isolated normal glomeruli [57]. The data suggested that NF-κB was spontaneously activated in explanted glomeruli via autocrine/paracrine factors including TNFα.

Although NF-κB serves itself as a primary transcription factor for TNFα, there are other factors and mechanisms which control TNFα mRNA synthesis, transcript stability, translation and TNFα protein secretion. TNFα gene regulation in activated T cells involves AP-1 transcription factors ATF-2 and c-Jun which cooperate with NFATp [58]. In macrophages, c-Jun and C/EBPß transcriptionally activate TNFα, however regulation by NF-κB was found stronger and independent of these factors [59]. Covert and colleagues [3], [4] proposed that the LPS-induced TNFα secretion is mediated by TRIF-dependent activation of IRF3. Stability of TNFα mRNA is signal-dependent; Deleault et al. demonstrated that simultaneous activation of both ERK and p38 inhibit tristetraprolin and stabilize TNFα mRNA [60]. Massive TNFα protein production requires ERK and p38 atop of NF-κB in mice with constitutively active IKKß [61]. In LPS-stimulated murine dendritic cells, MK2, effector kinase of p38 promotes TNFα translation [62]. Interestingly, in articular chondrocytes and skeletal muscles, TNFα stimulates the activation of three subclasses of MAPKs: ERKs, p38, and JNKs [63], [64]. This opens the possibility that in some cells TNFα autocrine regulation involves both NF-κB-induced TNFα transcription and MAPK pathway-driven TNFα translation.

Majority of mechanisms which increase TNFα mRNA stability and translation, discussed above, are induced by the LPS stimulation, which strongly activates MAPK pathways as well as NF-κB via MyD88 (early phase) and TRIF-dependent pathways (late phase), reviewed in [65], [66]. Xaus et al. showed that LPS induces apoptosis in macrophages via autocrine TNFα production, and this mechanism is suppressed in TNFR1-deficient mice [42]. Hao and Baltimore found that TNFα mRNA degradation is several-fold lower when TNFα is produced in response to LPS when compared to TNFα stimulation [67]. This explains why the LPS stimulation leads to the massive secretion of TNFα, which in turn may trigger autocrine signaling, leading to prolonged oscillations of NF-κB, observed recently in a fraction of LPS-stimulated cells [68].

Finally, we should mention that there exist other cytokines, in particular IL-1, which are NF-κB-responsive [69], and which in turn may activate NF-κB [70]. For the sake of simplicity and clarity, we neglect this positive feedback loop in the current study.

## Methods

**Figure 1:**
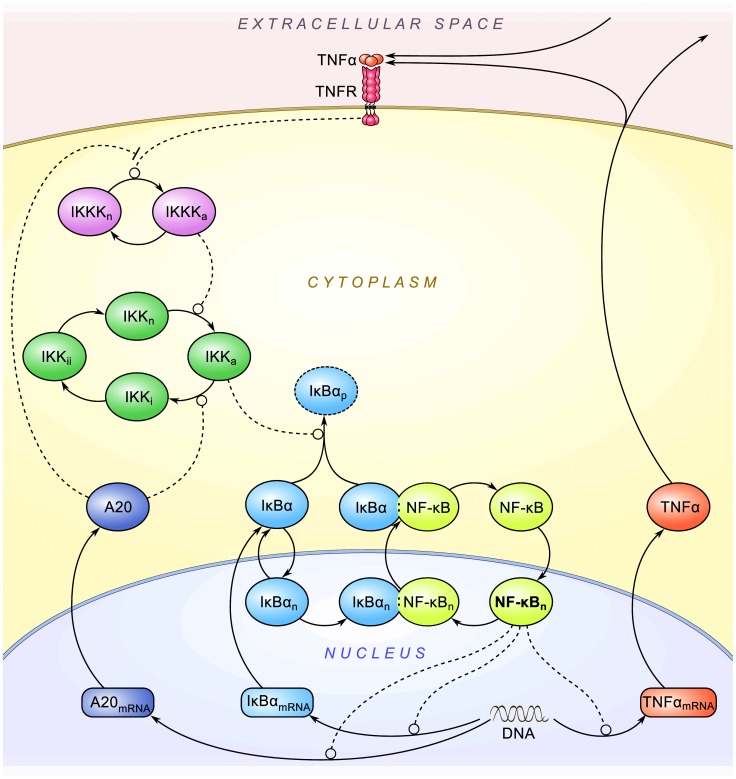
Schematic of the NF-κB model. Solid arrow-headed lines denote transitions; dashed lines denote influence: positive for circle-headed lines, negative for hammer-headed lines.

The modeling studies of NF-κB system started in 2002, by the study of oscillations of NF-κB–IκBα feedback loop (damped by the presence of IκBϵ and IκBß isoforms) by Hoffmann, Levchenko and colleagues [8], followed by Lipniacki et al. study introducing A20 regulatory loop [26], reviewed in [36], [71]. The considered model is based on our earlier studies [27], [34]. The key modification is the inclusion of autocrine regulation via TNFα which leads to the positive feedback loop and qualitatively changes dynamics of cells characterized by sizable TNFα synthesis. For completeness of the current study, we briefly review the structure of the model. The detailed description of the mathematical methods and the model, including the list of reactions and corresponding ordinary differential equations (ODEs), can be found in Text S1. The model involves seven proteins: NF-κB, its inducible inhibitors IκBα and A20, signal transduction kinases IKK and IKKK, cytokine TNFα and its receptor TNFR1 (Fig. 1). The model is two-compartmental and the translocations of NF-κB, IκBα and their complex between the cytoplasmic and nuclear compartments are considered. However, in contrast to recent studies by Terry and Chaplain [72], we do not account for spatial gradients (leading to the diffusion and transport terms) within these two compartments. Total levels of NF-κB, IKK and IKKK are assumed constant, without accounting for their production and degradation explicitly. In the case of IκBα, A20 and TNFα, the processes of mRNA transcription and protein translation are explicitly present in the model. The activation of corresponding genes follows NF-κB binding, while gene inactivation follows the NF-κB removal via IκBα binding.

### A20 And Iκbα Negative Feedback Loops

Nuclear NF-κB activity is controlled by two interlinked negative feedback loops, one mediated by IκB proteins: IκBα and IκB, the other mediated by A20 (Fig. 1). The inhibitors IκBα and IκB bind NF-κB and sequester it in the cytoplasm. Upon the signal mediated by the kinases IKKK and IKK, IκBα is phosphorylated and rapidly degraded. IκB is also phosphorylated and degraded, although its degradation (and further resynthesis) is delayed by about 45 min with respect to IκBα. Free NF-κB translocates to the nucleus and triggers transcription of its inhibitors, IκBα, IκB and A20. Synthesized IκBα and IκB translocate to the nucleus, bind NF-κB and convey it back to the cytoplasm. IκB is several fold less abundant than the primary inhibitor IκBα, and as demonstrated by experimental and computational studies the main impact of IκB on the system dynamic is in desynchronizing cells [15]. Although individual cell trajectories are very similar for IκB-deficient and wild-type cells, the latter are less synchronized, and therefore oscillations appear damped when averaged over population [15],[35]. In the current study, we focus on TNFα autocrine regulation and neglect the regulatory differences between IκB and IκBα and replace these two proteins by a more abundant IκBα. The (NF-κB:IκBα) complexes may circulate between the nucleus and cytoplasm, however since they mostly accumulate in cytoplasm, which is visible in unstimulated cells (for which majority of NF-κB is bound to IκBα and other isoforms) [33],[34],[73], we neglect the nuclear import term for (NF-κB:IκBα). Accumulation of IκBα protein is enabled by A20 which attenuates the strength of the extracellular signal (discussed in Introduction). First, A20 attenuates the activity of TNFR1 receptors (which is the consequence of A20-induced degradation of RIP – the key component of the receptor complex). Second, it enhances conversion of catalytically active IKK (IKK), into catalytically inactive form (IKK). Inactive kinase IKK spontaneously converts back to the neutral form IKK through the intermediate form IKK. It is worth noticing here, that Ashall et al. [73] in their model variant assumed that A20 inhibits conversion of IKK to neutral form IKK, rather than it enhances conversion of IKK to IKK.

### Autocrine Tnfα Regulation

**Figure 2:**
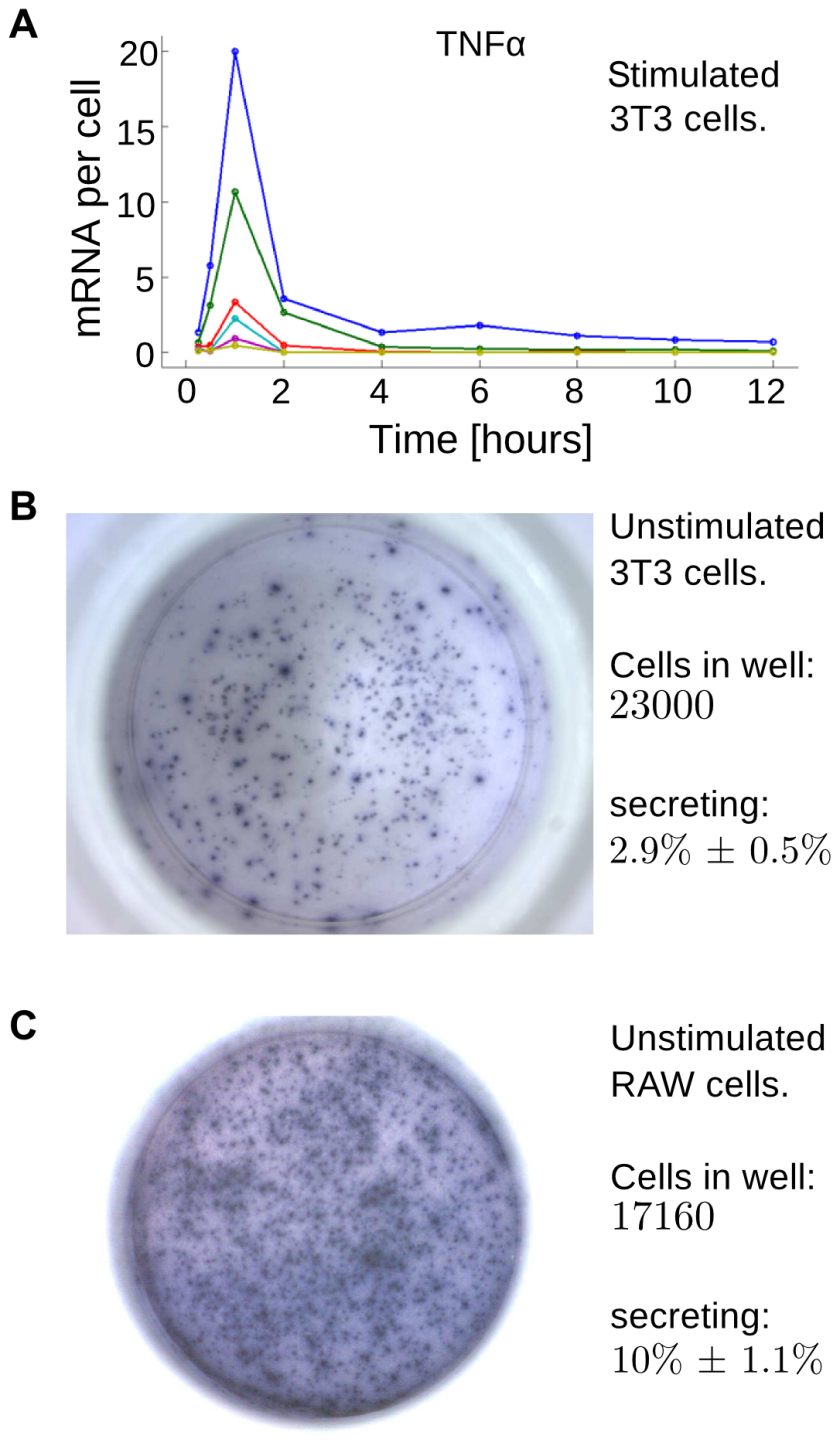
Evidence of TNFα synthesis and secretion in 3T3 cells and RAW cells. (**A**) Time-course of population averaged expression of TNFα mRNA in mouse 3T3 fibroblast cells stimulated with various doses of TNFα; color lines from dark blue to yellow correspond to TNFα doses of 10, 1, 0.1 0.05, 0.025 and 0.01 ng/ml. Cells were treated with different doses of TNFα, and TNFα mRNA was quantified at different times using microfluidic qPCR. Microfluidic digital-PCR was used to calibrate expression levels to mRNA counts. (**B**, **C**) Representative ELISpot assays showing TNFα secretion by (**B**) unstimulated 3T3 and (**C**) RAW cells.

TNFα is one of NF-κB-responsive genes, and its expression level is cell type-dependent. 3T3 cells, which we studied experimentally in this work, exhibit a relatively low TNFα expression, reaching 20 mRNA molecules (on average) per cell at the highest TNFα stimulation dose, Fig. 2A. However, expression levels calculated per activated cell were independent of the TNFα dose showing digital responses similar to that of early genes we analyzed earlier [34]. The dynamic gene expression measurements show that the TNFα synthesis has a distinct peak at *t*  =  1 hour regardless of the TNFα dose, and shows a low plateau which extends to beyond 10 hours.

Interestingly, we found that a small fraction (about 3%) of 3T3 cells secrete TNFα without any stimulation as shown by ELISpot assay, Fig. 2B. The fraction of secreting cells was found to be larger (about 10%) for RAW 264.7 (mouse leukaemic monocyte macrophage) cells, Fig. 2C. These measurements add to the evidence that TNFα production and secretion can be triggered spontaneously, and that probability of such spontaneous activations is cell line-dependent. Motivated by this observation, and earlier experimental studies demonstrating that TNFα induces TNFα synthesis via NF-κB activation ([51],[52] as discussed in Introduction), we expanded our earlier model to include the TNFα autocrine regulation. Accordingly, we consider NF-κB-inducible TNFα mRNA synthesis, followed by TNFα protein translation and secretion. We assume that some fraction of secreted TNFα molecules may bind to receptors on the same cell, and that the fraction of captured TNFα molecules increases with the number of TNFR1 receptors according to the Hill function. The fraction of secreted TNFα which is not bound by receptors of the secreting cell is neglected in the considerations, but could be accounted for by modifying the extracellular TNFα concentration.

We analyze the evolution of the NF-κB system in the absence of any stimulation as well as its responses to the imposed concentrations of TNFα, considering both tonic and pulsed stimulation. In the whole analysis we account for intracellular and extracellular TNFα degradation, with degradation half-time 1 h (degradation rate of s), consistent with our earlier estimations [34].

### Deterministic And Stochastic Modeling

We and others predicted and demonstrated that responses of the NF-κB system to low TNFα doses, as well as low LPS doses, are highly stochastic, and only a fraction of cells exhibit measurable NF-κB activation [27],[34],[74],[75]. Our ELISpot data on 3T3 cells and macrophages show that only a small fraction of cells secrete TNFα. Therefore, in order to analyze the autocrine TNFα regulation we will combine deterministic and stochastic modeling. In the deterministic approximation, the system of 25 ODEs is derived from the list of chemical reactions. The equations are then solved using Matlab and BioNetGen (Materials S1 and S2). The deterministic approximation is used to analyze the dynamical structure of the regulatory system, which is needed to properly interpret more complex stochastic trajectories. Based on the bifurcation analysis performed using MatCont continuation software (see Text S1 and Material S3), we will show that unstimulated wild-type (WT) cells may have, depending on the strength of the autocrine regulation, two stable recurrent solutions: steady state and limit cycle, the latter corresponding to the cytoplasmic–nuclear NF-κB oscillations. In contrast, A20-deficient cells may simultaneously have two stable steady state solutions, corresponding to the active and inactive cells.

In the stochastic approach, chemical reactions are simulated using the direct Stochastic Simulation Algorithm [76] implemented in BioNetGen. BioNetGen is a rule-based specification language and environment [77]. In BioNetGen language, models are constructed by specifying rules that describe allowed protein–protein interactions, processes, and covalent modifications. Based on the rules, the reaction network is automatically generated along with the system of ODEs. The advantage of this approach is that it allows for concise definitions of models with large numbers of interactions and protein states [78]. Here, the model is relatively small, and the BioNetGen software is used because of its very efficient implementation of the Stochastic Simulation Algorithm (direct method). Trajectories obtained in stochastic simulations are interpreted as single cell trajectories. These trajectories, as we will see, may switch between the attractors of the deterministic approximation or may exhibit the excitatory behavior. Stochastic simulations will be used to determine the fraction of responding cells as a function of the TNFα dose. Averages over a large number of stochastic trajectories will be used to fit the model to the population data. As demonstrated before, in non-linear systems, the average over a large number of stochastic trajectories may qualitatively differ from the trajectory obtained in the deterministic approximation, and thus the deterministic approximation of the process may not satisfactorily reproduce population data [79]. In the stochastic model two types of noise are considered:

#### Extrinsic Noise

The analysis performed in our previous study [34] indicated for a broad distribution of TNFR1 receptor number across the cell population. The heterogeneity of NF-κB expression is of smaller importance, and will be neglected here for the sake of simplicity. Following [34] we assume that the number of receptors is log-normally distributed with probability density (see also Fig. S1 in Text S1),with and . Such distribution is characterized by median , mean and variance . In the deterministic approximation, if not otherwise specified, we assumed that the number of receptors is equal to median .

#### Intrinsic Noise

Intrinsic noise in the system results mainly from the discrete regulation of TNFR1 receptors activity and activation of A20, IκBα, and TNFα genes, see [36],[80]. We found, however, that at low or zero dose stimulation, when the number of A20, IκBα, and TNFα mRNA molecules is very low, the transcriptional noise is also important. Accordingly, in contrast to our earlier studies [27],[34],[35] that relied on Haseltine and Rawlings algorithm [81], in the current study we perform all stochastic simulations using the direct method of Gillespie [76].

## Results

### Analysis Of The Deterministic Model

#### Wild-Type Cells

**Figure 3:**
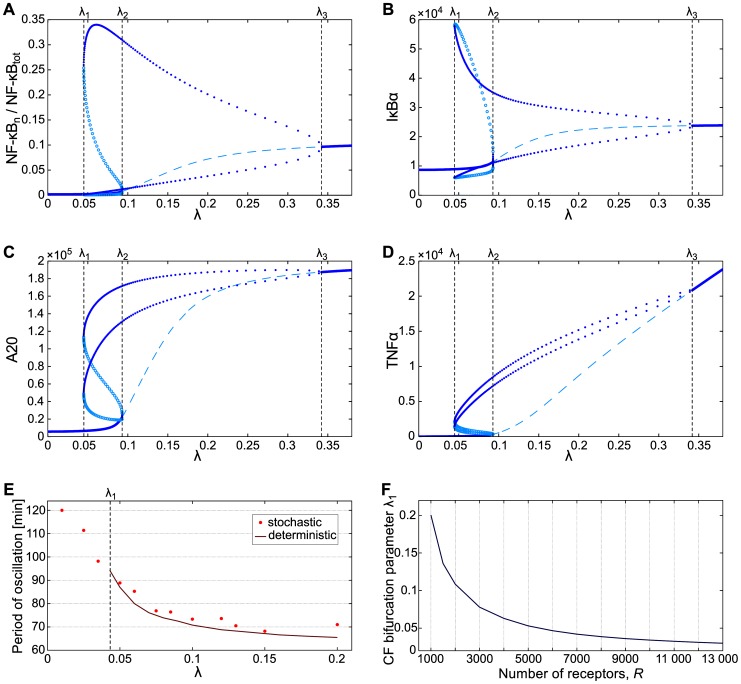
Bifurcation diagrams for WT cells. Recurrent solutions in a function of TNFα mRNA transcription coefficient . (**A**–**D**) Nuclear NF-κB, free cytoplasmic IκBα, A20, intracellular TNFα. There are three bifurcations: cyclic fold (CF) at , subcritical Hopf at (see Text S1 and Fig. S3 therein for details) and supercritical Hopf at . (**E**) Oscillation period of stochastic and deterministic trajectories as a function of . (**F**) Cyclic fold bifurcation parameter as a function of TNFR1 receptor number. Bifurcations diagrams shown in (**A**–**D**) where obtained for receptor number (equal to the median receptor number assumed for stochastic simulations).

The presence of the negative feedback loop together with the delay introduced by the mRNA transcription, protein translation and cytoplasmic to nuclear transport induces oscillatory responses to tonic TNFα stimulation. One can thus expect that cells which produce and secrete TNFα can exhibit tonic oscillations even without any external stimulation, being constantly activated by TNFα they secrete. In the bifurcation analysis (Fig. 3; see also Text S1, Fig. S3 for a 3-D plot), we consider the system without any external stimulation, i.e. assuming that the extracellular TNFα concentration equals zero. As a bifurcation parameter we choose TNFα mRNA synthesis rate , i.e., mRNA synthesis from a single active TNFα gene copy. The analysis shows that until the TNFα synthesis rate remains low, , the system may not exhibit persistent (limit cycle) oscillations. The only recurrent solution is the stable steady state in which the nuclear NF-κB fraction is low (below 0.01). At mRNA/s, the stable limit cycle arises in the cyclic fold bifurcation, and for intermediate TNFα synthesis rates, , the oscillatory solution coexist with the stable steady state solution. The further growth of the TNFα synthesis rate causes that the stable steady state solution loses its stability in bifurcation at mRNA/s, and in a broad range of the stable limit cycle is the only stable recurrent solution. A scrupulous analysis of the bifurcation at showed that in the very close vicinity of there are in fact two bifurcations: supercritical Hopf at and cyclic fold at (see Text S1, Fig. S2). These two bifurcations in coarse-grained view are equivalent to the single subcritical Hopf bifurcation and in further discussion will be considered as such. Finally, at mRNA/s the limit cycle oscillations are replaced by a single stable steady state. We should notice, however, that mRNA/s exceeds the physiological maximum transcriptional rate estimated as mRNA/s, assumed in the model for A20 and IκBα, known for very rapid mRNA synthesis (see [35]). In summary, we found that within the deterministic approximation, in the absence of stimulation, WT cells remain in the inactive state when TNFα synthesis rate is low (), or exhibit limit cycle oscillations for the high TNFα synthesis rate (). For intermediate TNFα synthesis rates cells may either remain in the inactive state or exhibit limit cycle oscillations. The values of bifurcation points, in particular at which limit cycle oscillations arise, decrease (almost linearly for small TNFR1 numbers) with cell sensitivity which is proportional to the assumed level of TNFR1 receptors, Fig. 3F.

#### A20-Deficient Cells

**Figure 4:**
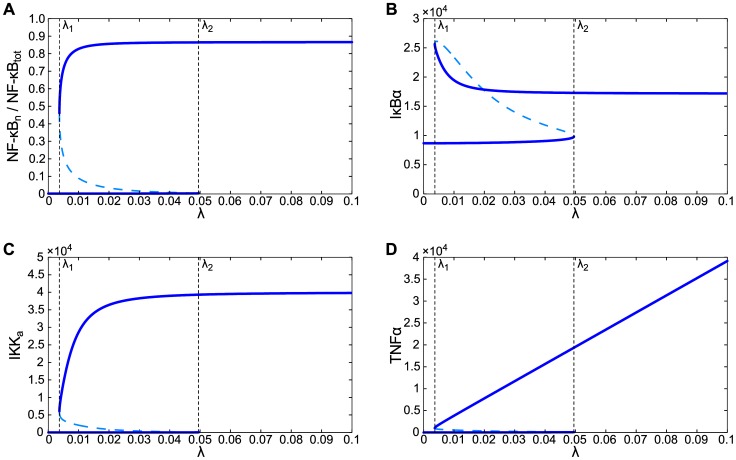
Bifurcation diagrams for A20-deficient cells. Stable recurrent solutions in a function of TNFα mRNA transcription coefficient . (**A**–**D**) Nuclear NF-κB, free cytoplasmic IBα, active IKK, intracellular TNFα. There are two saddle-node bifurcations at and.

In A20 cells the negative feedback is disturbed. Since A20 promotes transformation from active IKK (IKK) to inactive IKK (IKK), lack of A20 results in the prolonged IKK activity. This in turn prevents the accumulation of IκBα protein and results in the persistent nuclear NF-κB occupancy. As a result, in response to the tonic TNFα stimulation, A20-deficient cells do not exhibit limit cycle oscillation, but reach the active steady state, characterized by a high IKK activity, a high level of nuclear NF-κB and correspondingly high level of IκBα transcript, but low level of IκBα protein, which is constantly degraded due to the high IKK activity. One can thus expect that A20 cells which synthesize and secrete TNFα may remain in the active state, without external stimulation. In fact, the bifurcation analysis (Fig. 4) demonstrated that there exists a broad range of TNFα mRNA synthesis rate , , in which the system is bistable, i.e., it can remain either in the active state (with high nuclear NF-κB level) or the inactive state (with low nuclear NF-κB level). The value of parameter mRNA/s in which the active steady state appears (in saddle-node bifurcation) is very low, more than 10 times lower than the value of bifurcation parameter in which limit cycle oscillations arise in WT cells. The value of the second saddle-node bifurcation, , in which the inactive steady state vanishes, is much larger, mRNA/s. As a result, one may expect that A20-deficient cells will remain inactive without any stimulation, but even transient TNFα stimulation will drive them to the active state, in which they can remain for a long time (formally, infinitely long time).

The bifurcation analysis of the deterministic model demonstrated that due to the positive feedback regulation, even in the absence of any external stimulation, WT cells exhibit limit cycle oscillations while A20-deficient cells exhibit persistent activation, provided that TNFα mRNA synthesis rate is sufficiently large. The A20 cells were found to be much more sensitive, i.e., they can remain active for 10 times lower TNFα synthesis rate than needed for WT cells activation. In addition, we found that both A20 and WT cells exhibit bistability: in WT cells it is manifested by the coexistence of the stable limit cycle and the stable steady state. One can thus expect that real (noisy) cells will exhibit transitions between the basins of attraction of recurrent solutions found in the deterministic analysis.

### Stochastic Switching In The Absence Of Tnfα Stimulation

**Figure 5:**
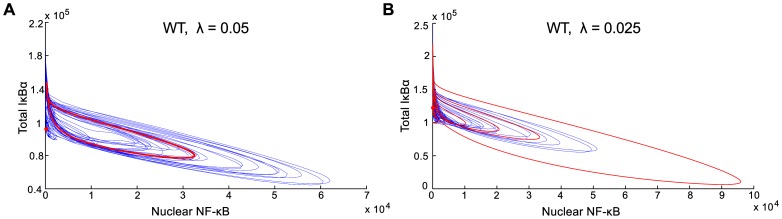
Stochastic versus deterministic solutions for WT cells. (**A**) ; thick red line and red dot – stable limit cycle and stable steady state for the deterministic approximation; blue line – example stochastic trajectory (total simulation time: 70 h). (**B**) ; red line – deterministic damped oscillations in response to 5-min pulsed 1 ng/ml TNFα; blue line – example stochastic trajectory (total simulation time: 70 h) in the absence of TNFα stimulation.

In Fig. 5 we compare deterministic and stochastic trajectories projected onto the (‘Nuclear NF-κB’, ‘Total IκBα’) plane. For mRNA/s (Fig. 5A) the system in the deterministic approximation has the stable steady state and the stable limit cycle. As expected, the stochastic trajectory switches between limit cycle oscillations and small fluctuations in the vicinity of the inactive steady state. The large magnitude of noise causes large departures from the stable orbit of the deterministic approximation. For the twice smaller value of mRNA/s (Fig. 5B), the inactive steady state is the only recurrent solution of the deterministic system. The deterministic trajectory (red line), after the large departure from this unique stable steady state in response to the 5-min 1 ng/ml TNFα pulse, exhibits a series of four oscillations before returning to the close vicinity of the steady state. In contrast, a stochastic trajectory may exhibit longer series of semi-periodic oscillations, without any TNFα stimulation (black line). The phenomenon of noise-induced oscillations is quite common in dynamical systems; here, the oscillations are additionally stabilized by the “ghost” of the limit cycle.

**Figure 6:**
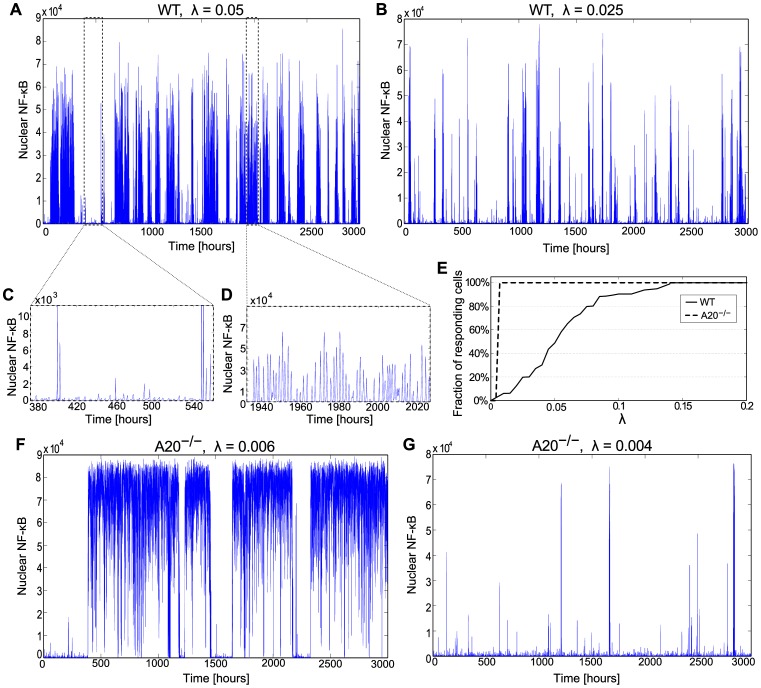
Long run stochastic trajectories in the absence of external stimulation. (**A**, **B**) WT cells for  = 0.05 and  = 0.025, respectively, and TNFR1 receptors number  = 7000. (**C**, **D**) Zoomed fragments of trajectory showing (**C**) small stochastic fluctuations in the vicinity of the stable steady state and (**D**) large amplitude oscillations in the basin of attraction of the stable limit cycle. (**E**) Fraction of oscillating WT and A20 cells as a function of . (**F**, **G**) A20 cells trajectories for  = 0.006 and  = 0.004.

In Fig. 6 we analyze stochastic switching of WT and A20 cells. WT cells are analyzed for two TNFα transcription coefficients mRNA/s (Fig. 6A) and mRNA/s (Fig. 6B). In the first case, 3000-hour-long simulation reveals irregular jumps between the inactive and the oscillatory phases (Fig. 6A). In the inactive phase (Fig. 6C), the nuclear NF-κB fluctuations are irregular and their amplitude is of order of molecules. In contrast, in the oscillatory phase, the oscillations are semiperiodic with the average amplitude of molecules (Fig. 6D), more than an order of magnitude larger than in the inactive phase. For the stochastic transitions between the inactive and the oscillatory phases occur on average every 70 h, and the fraction of time spent in each phase is almost equal. For smaller mRNA/s, for which the deterministic system is monostable, transitions to the oscillatory phase are still possible, but the characteristic number of oscillations in a series is smaller. As one could expect, the probability that a cell is in the oscillatory phase grows with (Fig. 6E). A bit surprisingly, even when the deterministic approximation is monostable ( mRNA/s), the oscillatory phase probability is nonzero, and, similarly, when the deterministic systems has only limit cycle oscillations ( mRNA/s), the oscillatory phase probability may still be smaller than 1.

As already said, A20-deficient cells are more sensitive to TNFα, and they are activated at a much smaller TNFα transcription coefficient . This property is even more evident when the stochastic system is analyzed. For the transitions to the active state are very infrequent (Fig. 6G), but for larger cells spend more than half of time in the active state. Despite the deterministic system is bistable for , it appears that the stochastic system is persistently active for (Fig. 6E and Text S1, Fig. S4C).

### Individual Cell Responses To Different Tnfα Doses

#### Wild-Type Cells

**Figure 7:**
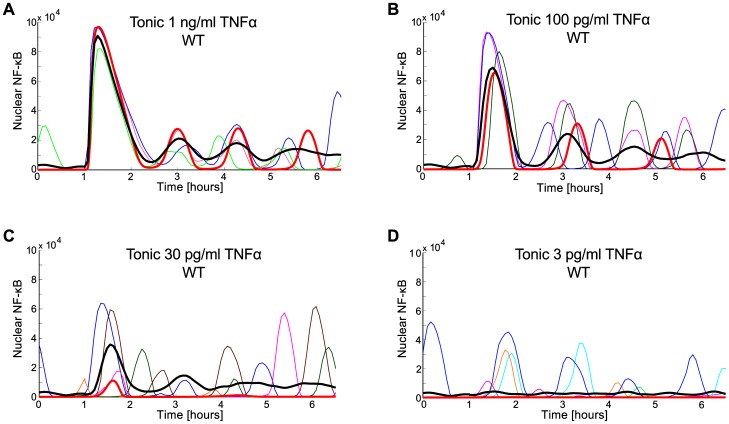
Simulated responses of WT cells (with  = 0.025) to tonic TNFα stimulation beginning at  = 1 h. (**A**–**D**): TNFα doses: 1 ng/ml, 100 pg/ml, 30 pg/ml, 3 pg/ml. Red thick line – deterministic simulation; thin colored lines – single cell stochastic simulations; black thick line – population average. In (**A**) and (**B**), 3 individual representative cells trajectories are shown (in each panel). In (**C**) and (**D**), respectively 5 and 10 individual cell trajectories are shown, but only 3 trajectories (in each panel) exhibit visible oscillations.

Turner et al. [75] found that about 20% of unstimulated SK-N-AS cells exhibit NF-κB oscillations without any stimulation. In light of our model, this finding suggests that these cell express TNFα, and that the TNFα transcription coefficient, , is about mRNA/s (or, more precisely, that effectiveness of TNFα transcription, translation and secretion process is such as in the model for mRNA/s). As shown in Fig. 6E for this , the probability to find a cell in the oscillatory phase is about 20%. More precisely, the fitted value of , as well as the cyclic fold bifurcation parameter , depend on the assumed level of TNFR1 receptors (Fig. 3F). Keeping the experiment of Turner et al. as a reference for SK-N-AS cells, we set mRNA/s [75]. As shown in Fig. 3E for the oscillation period (of spontaneous oscillation) is about 110 min in agreement with experimental data, and then decreases with the value of . Accordingly, for we simulate cell responses to four TNFα doses. In simulations the level of TNFα is increased abruptly in h from 0 to respectively 1 ng/ml, 100 pg/ml, 30 pg/ml, 3 pg/ml, and then decreases exponentially with half-time of ∼1 h due to protein degradation (Fig. 7).

The single cell trajectories obtained in numerical simulations (Fig. 7) are in plausible agreement with the experiment of Turner et al. [75]. In particular, both experiment and simulation showed that the amplitude of the first pulse decreases with dose, but the amplitudes of subsequent peaks are higher for the low than for the high dose.

The low dose (≤30 pg/ml) responses have a purely stochastic nature. They are not observed in the deterministic simulations (thick red line), and are invisible at the population level due to the asynchrony of individual cells. The average activation time and its variance increases with decreasing TNFα dose, which suggests that the first activation has a stochastic character. As predicted and demonstrated recently, massive NF-κB translocation may follow binding of single TNFα molecules to TNFR1 receptors [27],[34]. However, even at low doses the first peak is frequently followed by subsequent ones, which according to the model is due to (1) autoactivation via autocrine TNFα regulation and (2) broad distribution of the level of receptors. Responding cells likely have higher receptor number so they are more prone for subsequent activation [34].

**Figure 8:**
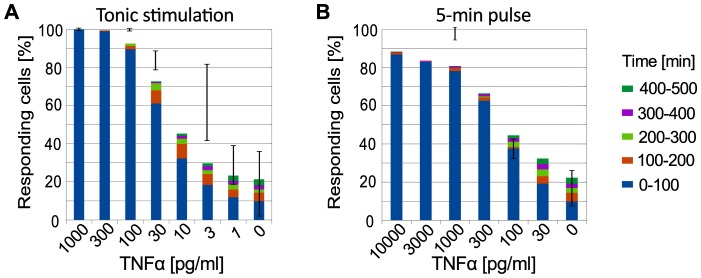
Fraction of responding cells versus TNF*α*dose. (**A**) Tonic stimulation. (**B**) 5-min pulsed stimulation. Color bars: model prediction for  = 0.025– fraction of cells responding within the given time period. Error bars show fractions of cells responding during the first 300-min in the experiment of Turner et al. [75] on SK-N-AS cells, see the main text.

As found by Turner et al., the fraction of activated cells in first 300 minutes decreases with TNFα dose, but even for zero doses the activated cell fraction is about 20% [75]. This phenomenon is clearly visible in our simulation (Fig. 8). Following Turner et al., we analyze two cases: tonic TNFα stimulation, and 5-min TNFα pulse [75]. As expected, for the same dose, tonic stimulation yield higher fraction of responding cells. The model predictions are in reasonable agreement with experiment, with the main difference being observed for the tonic stimulation. For 3 pg/ml the model predicts lower fraction of responding cells than that observed experimentally. This can be attributed to the paracrine activation of neighboring cells, which is not taken into account in the model. Paracrine signaling can be also responsible for huge error bars for 3 pg/ml dose: one can imagine that the denser arrays of cells are more prone to activation.

#### A20-Deficient Cells

In their seminal work, Lee et al. observed that A20 MEFs (in contrast to WT cells) do not exhibit oscillations to the tonic TNFα stimulation [14]. More surprisingly, Werner et al. (2005 and 2008) observed that in A20 MEFs even a short 5-min pulse of TNFα stimulation leads to at least 3-hour-long nuclear NF-κB activity [82],[83]. As already found in the deterministic model analysis, A20 cells producing even small amounts of TNFα are bistable, and thus may be “persistently” activated by a short pulse of TNFα.

**Figure 9:**
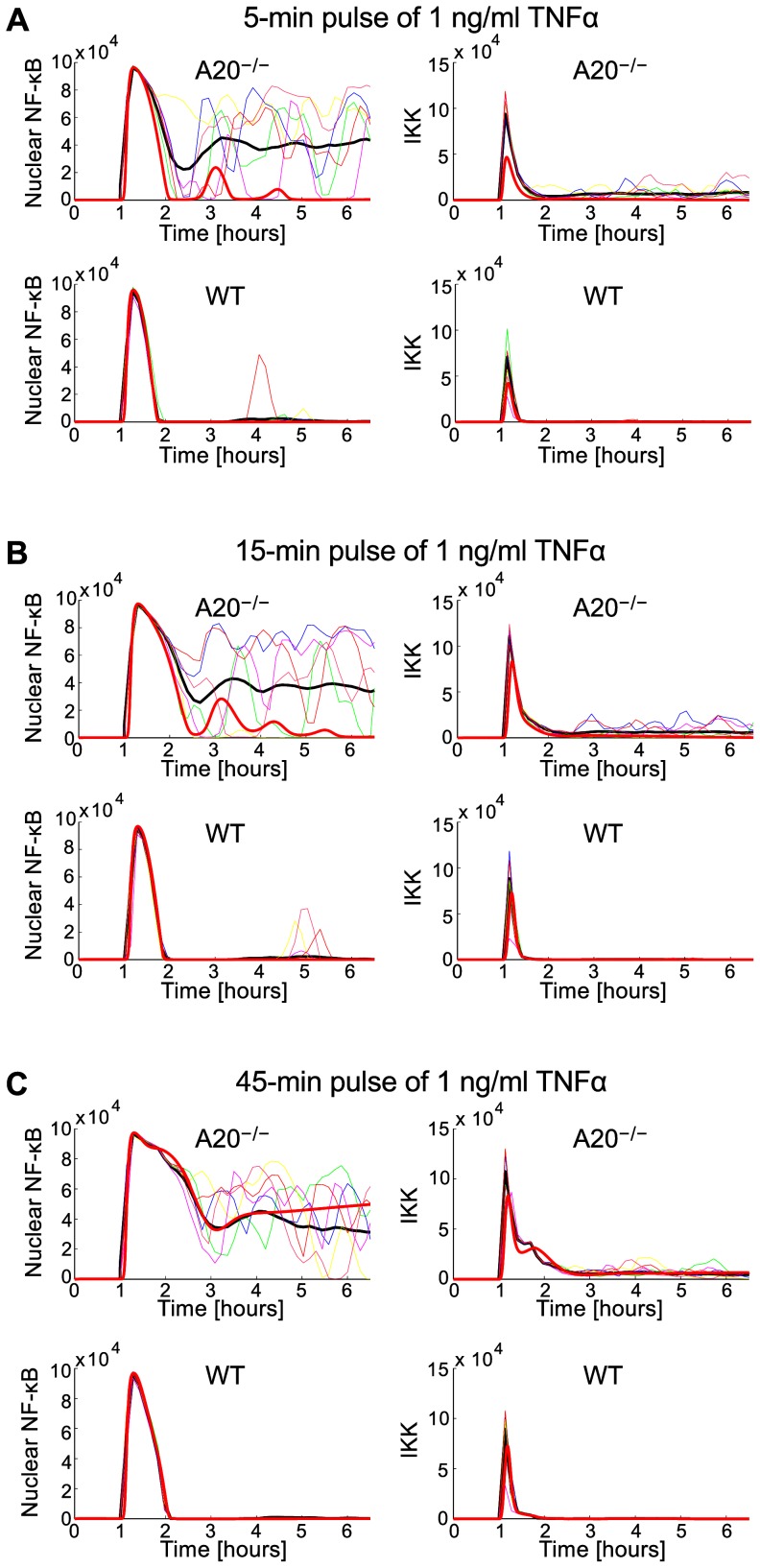
Simulated responses of WT and A20 cells to pulsed stimulation with 1 ng/ml TNFα for  = 0.004. (**A**) 5-min pulse. (**B**) 15-min pulse. (**C**) 45-min pulse. Red thick line – deterministic simulation; thin colored lines – single cell stochastic simulations; black thick line – population average.

In Fig. 9 we compare WT and A20 cell responses to 5-, 15- and 45-min TNFα stimulation. We assume TNFα mRNA synthesis rate , much smaller than the value for SK-N-AS cells. This is in accordance with the observation that 3T3 cells do not exhibit spontaneous activation. WT cells respond with a single pulse of IKK activity, which leads in most cases to a single pulse of nuclear NF-κB. In contrast, A20 cells show a high tail of IKK activity, which results in the prolonged nuclear NF-κB occupancy. In the deterministic model (thick red line), 5- and 15-min pulses are not sufficient to drive cells into the active state; only after the 45-min pulse cells became persistently activated. In contrast, most of single cell stochastic trajectories exhibit a high level of nuclear NF-κB even after the 5-min pulse. As a result, the population average trajectory shows single NF-κB pulse followed by a very high tail. The IKK and NF-κB activity profiles for WT and A20 are in plausible agreement with experiments of Werner et al. [82],[83].

## Discussion

We investigated theoretically and computationally the effect of autocrine TNFα signaling on NF-κB regulation. NF-κB activity is regulated by two interlinked negative feedback loops. The first loop involves NF-κB responsive inhibitors: IκBα and IκB, which directly bind to NF-κB and sequester it in the cytoplasm. The second loop is mediated by another NF-κB strongly responsive protein, A20, which attenuates the IKK activity. Without A20 expression, IKK retains its activity, which leads to the rapid degradation of the newly synthesized IκBα and destroys the NF-κB–IκBα feedback loop. The autocrine positive feedback loop arises in cell lines that are characterized by a sufficiently high TNFR1 expression and TNFα secretion. As demonstrated in this study, the positive feedback qualitatively changes the system dynamics. It may lead to long-lasting NF-κB oscillations in WT cells and persistent NF-κB activity in A20-deficient cells, which were found to be very prone to activation. The approach proposed in this study combined deterministic and stochastic modeling.

Bifurcation analysis was performed for WT and A20-deficient cells. In both cases, TNFα mRNA synthesis rate was chosen as a bifurcation parameter . Analysis of WT cells shown in Fig. 3 revealed that at some value of the limit cycle oscillations appear. These oscillations coexist with steady state (characterized by low level of nuclear NF-κB), which loses stability for . That is, in range of bifurcation parameter the system has two stable recurrent solutions, steady state and limit cycle. In contrast to WT cells, A20-deficient cells (considered in the deterministic approximation) do not exhibit oscillations. Instead, in a broad range of bifurcation parameter they exhibit bistability characterized by the coexistence of states of the low and high level of nuclear NF-κB. The A20 deficiency dramatically increases cell sensitivity: the critical value of TNFα synthesis at which cells may be activated due to autocrine signaling was found more than 10 times lower for A20 cells than for WT cells, ∼0.0037 mRNA/s (for A20) versus ∼0.045 mRNA/s (for WT).

By analyzing the stochastic model we demonstrated that noise, arising mostly at the level of gene regulation, enables switching between the stable steady state and limit cycle in WT cells, and between inactive and active steady state in A20-deficient cells (Fig. 6). Interestingly, in WT cells the semiperiodic oscillations can be driven by noise even for , i.e., in the absence of the limit cycle (Fig. 6E). This can be interpreted as *stochastic resonance*, which in the broad definition refers to the case when noise has a positive role in the signal-processing context [84]. The transition from the inactive to the oscillatory state can be also induced by an external TNFα stimulation, and the probability of such transition increases with the stimulation dose (Figs. 7 and 8). Based on our analysis, one should also expect that the LPS stimulation leading to the activation of NF-κB (which controls TNFα transcription) and MAPK pathways (effector kinases which stabilize TNFα transcript and enhance TNFα translation), which together results in massive secretion of TNFα, can also trigger long-lasting NF-κB oscillations in cells with high autocrine potential [4],[68].

Introduction of positive feedback enabled us to reproduce the noise-triggered oscillations observed by Turner et al. in unstimulated cells, as well as earlier experiments by Werner et al. showing prolonged NF-κB activation in response to the pulsed TNFα stimulation in A20-deficient MEFs [75],[82],[83]. Since the sensitivity to the autocrine-driven activation of A20-deficient cells is much higher than that of WT cells, even a weak stimulus can drive these cells to the state of persistent NF-κB activation characterized by massive secretion of TNFα and other inflammatory cytokines such as IL-8 and IL-6. This explains why the loss of A20 or its dysfunction disturbs regulation of immune system and renders the organism vulnerable to the septic shock resulting from the uncontrolled secretion of inflammatory cytokines [85]. Mice lacking A20 are hypersensitive to the TNFα-induced cell death, which suggests that positive auto- and paracrine signaling upregulate the TNFα expression so strongly that it overcomes the antiapoptotic action of NF-κB [14]. Boone et al. demonstrated that A20 is critical for the regulation of macrophage responses in vivo and protects mice against the septic shock [28].

There is a bulk of evidence that the loss or dysfunction of A20 as well as the other inhibitory DUBase, named Cyld, promote inflammatory diseases and cancer (reviewed in [24],[86]). It was found recently that A20 functions as a tumor suppressor in several subtypes of non-Hodgkin as well as Hodgkin lymphomas, and its silencing results in the constitutive activation of NF-κB [29],[87]. Kato et al. found that when re-expressed in a lymphoma-derived cell line with no functional A20 alleles, wild-type A20, but not mutant A20, resulted in the suppression of cell growth and induction of apoptosis, accompanied by downregulation of NF-κB activation [87]. Somatic mutations of A20 are associated with constitutive activation of NF-κB and poor overall survival in diffuse large B-cell lymphoma [88]. Huang et al. observed that the loss of A20 expression accompanies the oncogenic transformation of MEFs [89]. The above findings indicate that constitutive NF-κB activation, resulting form A20 dysfunction or increased TNFα autocrine potential (due to elevated TNFα and/or TNFR1 expression), in general promote cancer [90],[91]. In correspondence to our considerations, Bian et al. found that constitutively active NF-κB is required for the survival of S-type neuroblastic SH-EP1 and SK-N-AS cell lines [92].

As already said, particular cell lines are characterized by the high TNFα autocrine potential. Macrophages are generally considered as major TNFα producers, and are also highly TNFα-responsive. There is a growing evidence that macrophages require autocrine TNFα regulation for survival and differentiation [93]–[95]. In monocytes, sustained Nrf2 activation that protects cells from oxidative damage involves TNFα autocrine signaling [56].

In summary, the proposed model explains the mechanism of spontaneous or signal-dependent activation of NF-κB in cells with high autocrine potential. The cells prone to autocrine activation are characterized by high level of TNFα and TNFR1 synthesis or loss of functional A20. A20 dysfunction may promote inflammation and cancer, and also render the organism vulnerable to septic shock. In some cell lines, however, the self-sustained NF-κB activation can be required for performing their functions or undergo differentiation.

## Supporting Information

Text S1 **The supplementary text includes: list of reactions and parameters, list of differential equations, numerical simulation protocols, mathematical methods and experimental protocols, and four supplementary figures: Fig. S1– distribution of the number of receptors; Fig. S2– close-up on the bifurcation diagram near for WT cells; Fig. S3––3-D bifurcation diagram for WT cells; Fig. S4– long run stochastic trajectories for WT cells for *λ* = 0.1 and *λ* = 0.2 and for A20 cells for *λ* = 0.01.**
(PDF)

Material S1 **Matlab code of the model for performing deterministic simulations. (ZIP-archived directory containing Matlab scripts and a ReadMe file).**
(ZIP)

Material S2 **BioNetGen code of the model for performing both deterministic and stochastic simulations. (ZIP-archived directory containing a BNGL model file and a ReadMe file).**
(ZIP)

Material S3 **Matlab/Matcont code for performing bifurcation analysis. (ZIP-archived directory containing Matlab scripts calling Matcont functions, and a ReadMe file).**
(ZIP)
