# Supplementary material for: Spontaneous NF-κB Activation by Autocrine TNFα Signaling: A Computational Analysis
Source: PLoS One. 2013 Nov 11;8(11):e78887. doi: 10.1371/journal.pone.0078887 (PMC3855823; doi:10.1371/journal.pone.0078887)
Supplement: Text S1 — The supplementary text includes: list of reactions and parameters, list of differential equations, numerical simulation protocols, mathematical methods and experimental protocols, and four supplementary figures: Fig. S1– distribution of the number of receptors; Fig. S2– close-up on the bifurcation diagram near for WT cells; Fig. S3––3-D bifurcation diagram for WT cells; Fig. S4– long run stochastic trajectories for WT cells for λ = 0.1 and λ = 0.2 and for A20 cells for λ = 0.01. (PDF) [file pone.0078887.s001.pdf]

Spontaneous NF-κB Activation by Autocrine TNFα Signaling: A Computational Analysis

Jakub Pękalski, Pawel J. Zuk, Marek Kochańczyk,
Michael Junkin, Ryan Kellogg, Savaş Tay,
and Tomasz Lipniacki

Supporting Information

Supplementary Text S1 with Figures S1–S4

1 Parameters and reactions

Table — Notation guide

Symbol	*Description*
$TNFR_{\mathrm{i}}$	inactive TNFR1 receptors
$TNFR_{\mathrm{a}}$	active TNFR1 receptors
$IKKK_{\mathrm{n}}$	neutral form of IKKK
$IKKK_{\mathrm{a}}$	active form of IKKK
IKK_{i}	inactive form of IKK
IKK_{ii}	inactive intermediate form of IKK
IKK_{n}	neutral form of IKK kinase
IKK_{a}	active form of IKK
$A20_{\mathrm{mRNA}}$	A20 transcript
$A20$	cytoplasmic A20
$I\kappa B\alpha_{\mathrm{mRNA}}$	IκBα transcript
$I\kappa B\alpha$	cytoplasmic IκBα
$I\kappa B\alpha_{\mathrm{n}}$	nuclear IκBα
$I\kappa B\alpha_{\mathrm{p}}$	phosphorylated cytoplasmic IκBα
$NF\kappa B$	cytoplasmic NF-κB
$NF\kappa B_{\mathrm{n}}$	nuclear NF-κB
$(NF\kappa B : I\kappa B\alpha)$	cytoplasmic NF-κB:IκBα complexes
$(NF\kappa B_{\mathrm{n}} : I\kappa B\alpha_{\mathrm{n}})$	nuclear NF-κB:IκBα complexes
$(NF\kappa B : I\kappa B\alpha_{\mathrm{p}})$	phosphorylated cytoplasmic IκBα complexed to NF-κB
TNF_{mRNA}	TNFα transcript
TNF	intracellular TNFα
TNF_{ext}	extracellular TNFα
$G^i_{\mathrm{I\kappa B}}$	state of the ith IκBα gene copy, discrete random variable: $G^i_{\mathrm{I\kappa B}} \in \{0, 1\}$
G^i_{A20}	state of the ith A20 gene copy, discrete random variable: $G^i_{\mathrm{A20}} \in \{0, 1\}$
G^i_{TNF}	state of the ith TNFα gene copy, discrete random variable: $G^i_{\mathrm{TNF}} \in \{0, 1\}$
$G_{\mathrm{I\kappa B}}$	$G_{\mathrm{I\kappa B}} := \sum_i G^i_{\mathrm{I\kappa B}}$
G_{A20}	$G_{\mathrm{A20}} := \sum_i G^i_{\mathrm{A20}}$
G_{TNF}	$G_{\mathrm{TNF}} := \sum_i G^i_{\mathrm{TNF}}$

Table — The cell parameters

Parameter	*Symbol*	*Value*	*Remarks*	*References*
$(\text{N:C ratio})^{-1} = \frac{\text{Volume of the cytoplasm}}{\text{Volume of the nucleus}}$	k_v	5	—	[2]
Number of IκBα gene copies	N_I	2	—	[1]
Number of A20 gene copies	N_A	2	—	[1]
Number of TNFα gene copies	N_T	2	—	this study
Median number of receptors	M_0	7×10^3	*(see also Figure S1, page 7)*	this study
Mean number of receptors	—	$\simeq 10^4$	$\text{mean} = M_0\, \mathrm{e}^{M_1^2/2},\, M_1 = \sqrt{0.7}$	this study
Variance of the number of receptors	—	$\simeq 10^8$	$\text{variance} = M_0^2\, (\mathrm{e}^{M_1^2} - 1)\, \mathrm{e}^{M_1^2}$	this study
Number of receptors	R	M_0	$R = \mathit{TNFR}_\mathrm{a}(t) + \mathit{TNFR}_\mathrm{i}(t)$	this study
Number of IKKK molecules	K_N	10^5	$K_\mathrm{N} = \mathit{IKKK}_\mathrm{n}(t) + \mathit{IKKK}_\mathrm{a}(t)$	[1]
Number of IKK molecules	K_NN	2×10^5	$K_\mathrm{NN} = \mathit{IKK}_\mathrm{n}(t) + \mathit{IKK}_\mathrm{a}(t) + \mathit{IKK}_\mathrm{i}(t) + \mathit{IKK}_\mathrm{ii}(t)$	[2]
Number of NF-κB molecules	$\mathit{NF\kappa B}_\mathrm{tot}$	10^5	$\mathit{NF\kappa B}_\mathrm{tot} = \mathit{NF\kappa B}(t) + \mathit{NF\kappa B}_\mathrm{n}(t) + (\mathit{NF\kappa B}:\mathit{I\kappa B\alpha})(t) + (\mathit{NF\kappa B}_\mathrm{n}:\mathit{I\kappa B\alpha}_\mathrm{n})(t) + (\mathit{NF\kappa B}:\mathit{I\kappa B\alpha}_\mathrm{p})(t)$	[2]

(For references, see page 6.)

Table — List of reactions

(See on the next page.)

Reaction	*Rate*	*Coefficients*	*Value*	*References*
TNFR1 activation and signal transduction cascade				
$TNF_{\mathrm{ext}} \to \varnothing$	c_{deg}	c_{deg}	2×10^{-4} s^{-1}	[1]
$TNFR_{\mathrm{i}} \to TNFR_{\mathrm{a}}$	$k_{\mathrm{b}} \cdot TNF_{\mathrm{ext}}$	k_{b}	1.2×10^{-5} s^{-1}(ng/ml)$^{-1}$	[1]
$TNFR_{\mathrm{i}} + TNF \to TNFR_{\mathrm{a}}$	$\dfrac{c_{\mathrm{sec}}}{TNFR_{\mathrm{i}} + c_{\mathrm{b}}}$	c_{sec}<br>c_{b}	10^{-5} s^{-1}<br>10^{4}	this study<br>this study
$TNFR_{\mathrm{a}} \to TNFR_{\mathrm{i}}$	k_{f}	k_{f}	1.2×10^{-3} s^{-1}	[1]
$IKKK_{\mathrm{n}} \to IKKK_{\mathrm{a}}$	$\dfrac{k_{\mathrm{a}} \cdot k_{\mathrm{A20}}}{k_{\mathrm{A20}} + A20} \cdot TNFR_{\mathrm{a}}$	k_{a}<br>k_{A20}	10^{-5} s^{-1}<br>10^{5}	this study<br>[1]
$IKKK_{\mathrm{a}} \to IKKK_{\mathrm{n}}$	k_{i}	k_{i}	10^{-2} s^{-1}	[1]
$IKK_{\mathrm{n}} \to IKK_{\mathrm{a}}$	$k_1 \cdot IKKK_{\mathrm{a}}^2$	k_1	6×10^{-10} s^{-1}	[1]
$IKK_{\mathrm{a}} \to IKK_{\mathrm{i}}$	$\dfrac{k_3}{k_2} \cdot (k_2 + A20)$	k_2<br>k_3	10^{4} s^{-1}<br>2×10^{-3} s^{-1}	[2]<br>[1]
$IKK_{\mathrm{i}} \to IKK_{\mathrm{ii}}$, $IKK_{\mathrm{ii}} \to IKK_{\mathrm{n}}$	k_4	k_4	10^{-3} s^{-1}	[1]
IκBα, A20 and TNFα gene expression				
$(G^i_{\mathrm{A20}} = 0) \to (G^i_{\mathrm{A20}} = 1)$<br>$(G^i_{\mathrm{I\kappa B\alpha}} = 0) \to (G^i_{\mathrm{I\kappa B\alpha}} = 1)$	$q_1 \cdot NF\kappa B_{\mathrm{n}}$	q_1	4×10^{-7} s^{-1}	[1]
$(G^i_{\mathrm{A20}} = 1) \to (G^i_{\mathrm{A20}} = 0)$<br>$(G^i_{\mathrm{I\kappa B\alpha}} = 1) \to (G^i_{\mathrm{I\kappa B\alpha}} = 0)$	$q_2 \cdot I\kappa B\alpha_{\mathrm{n}}$	q_2	10^{-6} s^{-1}	[2]
$(G^i_{\mathrm{TNF}} = 0) \to (G^i_{\mathrm{TNF}} = 1)$	$q_{1\mathrm{t}} \cdot NF\kappa B_{\mathrm{n}}$	$q_{1\mathrm{t}}$	4×10^{-8} s^{-1}	this study
$(G^i_{\mathrm{TNF}} = 1) \to (G^i_{\mathrm{TNF}} = 0)$	$q_{2\mathrm{t}} \cdot I\kappa B\alpha_{\mathrm{n}}$	$q_{2\mathrm{t}}$	10^{-6} s^{-1}	this study
$(G^i_{\mathrm{TNF}} = 1) \to (G^i_{\mathrm{TNF}} = 0)$	$q_{2\mathrm{tt}}$	$q_{2\mathrm{tt}}$	2×10^{-3} s^{-1}	this study
$\varnothing \to TNF_{\mathrm{mRNA}}$	$\lambda \cdot G^i_{\mathrm{TNF}}$	λ	*variable*	this study
$\varnothing \to A20_{\mathrm{mRNA}}$	$c_1 \cdot G^i_{\mathrm{A20}}$	c_1	0.1 s^{-1}	[2]
$A20_{\mathrm{mRNA}} \to \varnothing$	c_3	c_3	7.5×10^{-4} s^{-1}	[2]
$\varnothing \to A20$	$c_4 \cdot A20_{\mathrm{mRNA}}$	c_4	0.5 s^{-1}	[2]
$\varnothing \to I\kappa B\alpha_{\mathrm{mRNA}}$	$c_1 \cdot G^i_{\mathrm{I\kappa B\alpha}}$	c_1	0.1 s^{-1}	[2]
$I\kappa B\alpha_{\mathrm{mRNA}} \to \varnothing$	c_3	c_3	7.5×10^{-4} s^{-1}	[2]
$\varnothing \to I\kappa B\alpha$	$c_4 \cdot I\kappa B\alpha_{\mathrm{mRNA}}$	c_4	0.5 s^{-1}	[2]
$TNF_{\mathrm{mRNA}} \to \varnothing$	$c_{3\mathrm{t}}$	$c_{3\mathrm{t}}$	7.5×10^{-4} s^{-1}	this study
$\varnothing \to TNF$	$c_{4t} \cdot TNF_{\mathrm{mRNA}}$	$c_{4\mathrm{t}}$	0.05 s^{-1}	this study
Protein interactions and lifetime				
$NF\kappa B + I\kappa B\alpha \to (NF\kappa B\!:\!I\kappa B\alpha)$<br>$NF\kappa B_{\mathrm{n}} + I\kappa B\alpha_{\mathrm{n}} \to (NF\kappa B_{\mathrm{n}}\!:\!I\kappa B\alpha_{\mathrm{n}})$	a_1<br>$a_1 \cdot k_{\mathrm{v}}$	a_1<br>k_{v}	5×10^{-7} s^{-1}<br>5	[2]<br>[2]
$I\kappa B\alpha \to I\kappa B\alpha_{\mathrm{p}}$	$a_2 \cdot IKK_{\mathrm{a}}$	a_2	10^{-7} s^{-1}	[2]
$(NF\kappa B\!:\!I\kappa B\alpha) \to (NF\kappa B\!:\!I\kappa B\alpha_{\mathrm{p}})$	$a_3 \cdot IKK_{\mathrm{a}}$	a_3	5×10^{-7} s^{-1}	[2]
$\mathrm{A20} \to \varnothing$	c_5	c_5	5×10^{-4} s^{-1}	[2]
$I\kappa B\alpha_{\mathrm{p}} \to \varnothing$<br>$(NF\kappa B\!:\!I\kappa B\alpha_{\mathrm{p}}) \to NF\kappa B$	t_{p}	t_{p}	10^{-2} s^{-1}	[2]
$I\kappa B\alpha \to \varnothing$	$c_{5\mathrm{a}}$	$c_{5\mathrm{a}}$	10^{-4} s^{-1}	[2]
$TNF \to \varnothing$	$c_{\mathrm{sec}} + c_{5\mathrm{t}}$	c_{sec}<br>$c_{5\mathrm{t}}$	10^{-5} s^{-1}<br>2×10^{-4} s^{-1}	this study<br>this study
$(NF\kappa B\!:\!I\kappa B\alpha) \to NF\kappa B$	$c_{6\mathrm{a}}$	$c_{6\mathrm{a}}$	2×10^{-5} s^{-1}	[2]
Transport				
$NF\kappa B \to NF\kappa B_{\mathrm{n}}$	i_1	i_1	10^{-2} s^{-1}	[2]
$(NF\kappa B_{\mathrm{n}}\!:\!I\kappa B\alpha_{\mathrm{n}}) \to (NF\kappa B\!:\!I\kappa B\alpha)$	$e_{2\mathrm{a}}$	$e_{2\mathrm{a}}$	5×10^{-2} s^{-1}	[2]
$I\kappa B\alpha \to I\kappa B\alpha_{\mathrm{n}}$	$i_{1\mathrm{a}}$	$i_{1\mathrm{a}}$	2×10^{-3} s^{-1}	[2]
$I\kappa B\alpha_{\mathrm{n}} \to I\kappa B\alpha$	$e_{1\mathrm{a}}$	$e_{1\mathrm{a}}$	5×10^{-3} s^{-1}	[2]

2 Differential equations

$$\frac{\mathrm{d}}{\mathrm{d}t}TNFR_{\mathrm{a}}(t) = TNF(t)\cdot c_{\mathrm{sec}}\frac{TNFR_{\mathrm{i}}(t)}{TNFR_{\mathrm{i}}(t)+c_{\mathrm{b}}} + k_{\mathrm{b}}\cdot TNF_{\mathrm{ext}}(t)\cdot TNFR_{\mathrm{i}}(t) - k_{\mathrm{f}}\cdot TNFR_{\mathrm{a}}(t) \quad (1)$$

$$\frac{\mathrm{d}}{\mathrm{d}t}IKKK_{\mathrm{a}}(t) = k_{\mathrm{a}}\cdot\frac{k_{\mathrm{A20}}}{k_{\mathrm{A20}}+A20(t)}\cdot TNFR_{\mathrm{a}}(t)\cdot IKKK_{\mathrm{n}}(t) - k_{\mathrm{i}}\cdot IKKK_{\mathrm{a}}(t) \quad (2)$$

$$\frac{\mathrm{d}}{\mathrm{d}t}IKK_{\mathrm{a}}(t) = k_1\cdot IKKK_{\mathrm{a}}(t)^2\cdot IKK_{\mathrm{n}}(t) - IKK_{\mathrm{a}}(t)\cdot\frac{k_3}{k_2}(k_2+A20(t)) \quad (3)$$

$$\frac{\mathrm{d}}{\mathrm{d}t}IKK_{\mathrm{n}}(t) = -k_1\cdot IKKK_{\mathrm{a}}(t)^2\cdot IKK_{\mathrm{n}}(t) + k_4\cdot IKK_{\mathrm{ii}}(t) \quad (4)$$

$$\frac{\mathrm{d}}{\mathrm{d}t}IKK_{\mathrm{i}}(t) = IKK_{\mathrm{a}}(t)\cdot\frac{k_3}{k_2}(k_2+A20(t)) - k_4\cdot IKK_{\mathrm{i}}(t) \quad (5)$$

$$\frac{\mathrm{d}}{\mathrm{d}t}A20_{\mathrm{mRNA}}(t) = c_1\cdot G_{\mathrm{A20}}(t) - c_3\cdot A20_{\mathrm{mRNA}}(t) \quad (6)$$

$$\frac{\mathrm{d}}{\mathrm{d}t}A20(t) = c_4\cdot A20_{\mathrm{mRNA}}(t) - c_5\cdot A20(t) \quad (7)$$

$$\frac{\mathrm{d}}{\mathrm{d}t}I\kappa B\alpha_{\mathrm{mRNA}}(t) = c_1\cdot G_{\mathrm{I\kappa B\alpha}}(t) - c_3\cdot I\kappa B\alpha_{\mathrm{mRNA}}(t) \quad (8)$$

$$\begin{aligned}\frac{\mathrm{d}}{\mathrm{d}t}I\kappa B\alpha(t) = {} & -a_2\cdot IKK_{\mathrm{a}}(t)\cdot I\kappa B\alpha(t) - a_1\cdot I\kappa B\alpha(t)\cdot NF\kappa B(t) + c_4\cdot I\kappa B\alpha_{\mathrm{mRNA}}(t)\\ & -c_{5\mathrm{a}}\cdot I\kappa B\alpha(t) - i_{1\mathrm{a}}\cdot I\kappa B\alpha(t) + e_{1\mathrm{a}}\cdot I\kappa B\alpha_{\mathrm{n}}(t)\end{aligned} \quad (9)$$

$$\frac{\mathrm{d}}{\mathrm{d}t}I\kappa B\alpha_{\mathrm{n}}(t) = -a_1\cdot k_{\mathrm{v}}\cdot I\kappa B_{\mathrm{n}}(t)\cdot NF\kappa B_{\mathrm{n}}(t) + i_{1\mathrm{a}}\cdot I\kappa B\alpha(t) - e_{1\mathrm{a}}\cdot I\kappa B\alpha_{\mathrm{n}}(t) \quad (10)$$

$$\frac{\mathrm{d}}{\mathrm{d}t}I\kappa B\alpha_{\mathrm{p}}(t) = a_2\cdot IKK_{\mathrm{a}}(t)\cdot I\kappa B\alpha(t) - t_{\mathrm{p}}\cdot I\kappa B\alpha_{\mathrm{p}}(t) \quad (11)$$

$$\begin{aligned}\frac{\mathrm{d}}{\mathrm{d}t}NF\kappa B(t) = {} & c_{6a}\cdot(NF\kappa B\!:\!I\kappa B\alpha)(t) - a_1\cdot NF\kappa B(t)\cdot I\kappa B\alpha(t)\\ & +t_{\mathrm{p}}\cdot(NF\kappa B\!:\!I\kappa B\alpha_{\mathrm{p}})(t) - i_1\cdot NF\kappa B(t)\end{aligned} \quad (12)$$

$$\frac{\mathrm{d}}{\mathrm{d}t}NF\kappa B_{\mathrm{n}}(t) = i_1\cdot NF\kappa B(t) - a_1\cdot k_{\mathrm{v}}\cdot I\kappa B\alpha_{\mathrm{n}}(t)\cdot NF\kappa B_{\mathrm{n}}(t) \quad (13)$$

$$\begin{aligned}\frac{\mathrm{d}}{\mathrm{d}t}(NF\kappa B\!:\!I\kappa B\alpha)(t) = {} & a_1\cdot I\kappa B\alpha(t)\cdot NF\kappa B(t) - c_{6\mathrm{a}}\cdot(NF\kappa B\!:\!I\kappa B\alpha)(t)\\ & +e_{2\mathrm{a}}\cdot(NF\kappa B_{\mathrm{n}}\!:\!I\kappa B\alpha_{\mathrm{n}})(t) - a_3\cdot IKK_{\mathrm{a}}\cdot(NF\kappa B\!:\!I\kappa B\alpha)(t)\end{aligned} \quad (14)$$

$$\frac{\mathrm{d}}{\mathrm{d}t}(NF\kappa B\!:\!I\kappa B\alpha_{\mathrm{p}})(t) = a_3\cdot IKK_{\mathrm{a}}(t)\cdot(NF\kappa B\!:\!I\kappa B\alpha)(t) - t_{\mathrm{p}}\cdot(NF\kappa B\!:\!I\kappa B\alpha_{\mathrm{p}})(t) \quad (15)$$

$$\frac{\mathrm{d}}{\mathrm{d}t}TNF_{\mathrm{mRNA}}(t) = \lambda\cdot G_{\mathrm{TNF}}(t) - c_{3\mathrm{t}}\cdot TNF_{\mathrm{mRNA}}(t) \quad (16)$$

$$\frac{\mathrm{d}}{\mathrm{d}t}TNF(t) = c_{4\mathrm{t}}\cdot TNF_{\mathrm{mRNA}}(t) - c_{5\mathrm{t}}\cdot TNF(t) - c_{\mathrm{sec}}\cdot TNF \quad (17)$$

$$\frac{\mathrm{d}}{\mathrm{d}t}TNF_{\mathrm{ext}}(t) = -c_{\mathrm{deg}}\cdot TNF_{\mathrm{ext}} \quad (18)$$

$$\frac{\mathrm{d}}{\mathrm{d}t}G_{\mathrm{A20}}(t) = q_1\cdot NF\kappa B_{\mathrm{n}}(t)\cdot(N_{\mathrm{A}} - G_{\mathrm{A20}}(t)) - q_2\cdot I\kappa B\alpha_{\mathrm{n}}(t)\cdot G_{\mathrm{A20}}(t) \quad (19)$$

$$\frac{\mathrm{d}}{\mathrm{d}t}G_{\mathrm{I\kappa B\alpha}}(t) = q_1\cdot NF\kappa B_{\mathrm{n}}(t)\cdot(N_{\mathrm{I}} - G_{\mathrm{I\kappa B\alpha}}(t)) - q_2\cdot I\kappa B\alpha_{\mathrm{n}}(t)\cdot G_{\mathrm{I\kappa B\alpha}}(t) \quad (20)$$

$$\frac{\mathrm{d}}{\mathrm{d}t}G_{\mathrm{TNF}}(t) = q_{1\mathrm{t}}\cdot NF\kappa B_{\mathrm{n}}(t)\cdot(N_{\mathrm{T}} - G_{\mathrm{TNF}}(t)) - (q_{2\mathrm{tt}} + q_{2\mathrm{t}}\cdot I\kappa B\alpha_{\mathrm{n}}(t))\cdot G_{\mathrm{TNF}}(t) \quad (21)$$

....

$$\frac{\mathrm{d}}{\mathrm{d}t}TNFR_{\mathrm{i}}(t) = -TNF(t)\cdot c_{\mathrm{sec}}\frac{TNFR_{\mathrm{i}}(t)}{TNFR_{\mathrm{i}}(t)+c_{\mathrm{b}}} - k_{\mathrm{b}}\cdot TNF_{\mathrm{ext}}(t)\cdot TNFR_{\mathrm{i}}(t) + k_{\mathrm{f}}\cdot TNFR_{\mathrm{a}}(t) \quad (22)$$

$$\frac{\mathrm{d}}{\mathrm{d}t}IKKK_{\mathrm{n}}(t) = -k_{\mathrm{a}}\cdot\frac{k_{\mathrm{A20}}}{k_{\mathrm{A20}}+A20(t)}\cdot TNFR_{\mathrm{a}}(t)\cdot IKKK_{\mathrm{n}}(t) + k_{\mathrm{i}}\cdot IKKK_{\mathrm{a}}(t) \quad (23)$$

$$\frac{\mathrm{d}}{\mathrm{d}t}IKK_{\mathrm{ii}}(t) = k_4\cdot IKK_{\mathrm{i}}(t) - k_4\cdot IKK_{\mathrm{ii}}(t) \quad (24)$$

$$\frac{\mathrm{d}}{\mathrm{d}t}(NF\kappa B_{\mathrm{n}}\!:\!I\kappa B\alpha_{\mathrm{n}})(t) = a_1\cdot k_{\mathrm{v}}\cdot I\kappa B\alpha_{\mathrm{n}}(t)\cdot NF\kappa B_{\mathrm{n}}(t) - e_{2\mathrm{a}}\cdot(NF\kappa B_{\mathrm{n}}\!:\!I\kappa B\alpha_{\mathrm{n}})(t) \quad (25)$$

The last four variables from equations (22–25) can be determined equivalently by the following conservation laws:

$$\begin{aligned}
TNFR_{\rm i}(t) &= R - TNFR_{\rm a}(t) && (26)\\
IKKK_{\rm n}(t) &= K_{\rm N} - IKKK_{\rm a}(t) && (27)\\
IKK_{\rm ii}(t) &= K_{\rm NN} - IKK_{\rm a}(t) - IKK_{\rm i}(t) - IKK_{\rm n}(t) && (28)\\
(NF\kappa B_{\rm n} : I\kappa B\alpha_{\rm n})(t) &= NF\kappa B_{\rm tot} - NF\kappa B(t) - NF\kappa B_{\rm n}(t) - (NF\kappa B : I\kappa B\alpha)(t) - (NF\kappa B : I\kappa B\alpha_{\rm p})(t) && (29)
\end{aligned}$$

The complete system (1–25) is however more convenient for further development of the model.

3 Methods and protocols of numerical simulations

Deterministic model

The model was coded both in Matlab and BioNetGen. BioNetGen is a language and software intended for defining and simulating regulatory networks of high combinatorial complexity [3], and is capable of performing deterministic as well as stochastic simulations. BioNetGen allows for rule-based specification of the model; the rules are then used to build a list of reactions or ODEs which are solved by an integrated CVODE solver or simulated according to the Gillespie direct Stochastic Simulation Algorithm [4]. The model was originally written in Matlab and then rewritten to BioNetGen only to enable efficient stochastic simulations. Since the BioNetGen code results from the exact translation of the original Matlab code, the number of rules is equal to the number of reactions.

The bifurcation diagrams with TNFα transcription rate λ as the bifurcation parameter (Figs. 3 and 4 in the main text and Supplementary Figs. S2 and S3) were obtained using the MatCont continuation software. In order to perform the analysis, we used ODEs (1–21) in which variables $TNFR_{\rm i}$, $IKKK_{\rm n}$, $IKK_{\rm ii}$ and $(NF\kappa B_{\rm n} : I\kappa B\alpha_{\rm n})$ were defined by algebraic equations (26–29).

The simulations of WT and $A20^{-/-}$ cells showed in Figs. 5, 7 and 9 (in the main text) were started from the steady state corresponding to the extracellular TNFα concentration equal zero. $A20^{-/-}$ cells were modeled by setting the number of A20 gene copies equal to zero. Numerical integration was performed using the Matlab's implementation of the TR-BDF2 method (solver `ode23tb`).

Stochastic model

The stochastic simulations were performed using the Gillespie direct Stochastic Simulation Algorithm [4]. The direct method of stochastic simulation as implemented in BioNetGen allows for expressing reaction rates through functions of the system state, i.e. current number of molecules of given species. At every time step between consecutive reaction events, reaction propensities calculated using such functions remain constant. This enables exact simulation in accordance with the underlying Chemical Master Equation and at the same time allows for defining propensities not necessarily following the mass action kinetics.

To ensure random initial conditions at time $t = 0$, each stochastic simulation was started at time $t = -300$ h. The population average (Figs. 7 and 9 in the main text) was obtained based on 100 simulations. The fraction of responding cells (Fig. 6 in the main text) for each value of TNFα dose was obtained based on 500 simulations. For the purpose of analysis presented in Fig. 8 (in the main text), cells were considered activated when the fraction of nuclear NF-κB increased above 0.15, which allows for almost maximum expression of IκBα and A20 genes.

4 Experimental protocols

Gene expression analysis was performed on mouse 3T3 fibroblast cells using high-throughput microfluidic qPCR and digital-PCR. Cells grown on regular culture wells at equal density (80% confluence) were stimulated with various doses of TNFα at the beginning of experiments (10, 1, 0.1, 0.05, 0.025 and 0.01 ng/ml). At the end of each experiment (t = 0.25, 0.5, 2, 4, 6, 8, 10 and 12 hours), cells were lysed and cDNA was synthesized using standard protocols from Invitrogen (CellsDirect One-Step RT-PCR). Real-time qPCR was performed using the Invitrogen TaqMan probe for TNFα using the 48×48 Dynamic Array microfluidic chips and the Biomark System, both from Fluidigm. Each time and dose condition was repeated four times and the median value was used in Figure 2A (in the main text), resulting in a total of 192 qPCR measurements.

ELISpot experiments were carried out in a 96-well format according to manufacturer's instructions (R&D Systems ELISpot Kit EL410). NIH 3T3 fibroblast and 264.7 RAW macrophage cells were seeded onto plates at $\approx 2 \times 10^5$ cells/ml. The total number of cells added into each well was established via cell counting during seeding. After incubating cells for five hours, the assay was completed. Spots were manually counted from images taken with a Nikon SMZ 1500 microscope, without regard to intensity level of the spot. This allows for quantifying the percentage of cells which have secreted the cytokine over the course of the experiment.

Supplementary references

[1] Tay S, Hughey J, Lee T, Lipniacki T, Covert M, Quake M (2010) Single-cell NF-κB dynamics reveal digital activation and analogue information processing. *Nature* **466**:267–271.

[2] Lipniacki T, Puszynski T, Paszek P, Brasier AR, Kimmel M (2007) Single TNFα trimers mediating NF-κB activation: Stochastic robustness of NF-κB signaling. *BMC Bioinformatics* **8**:376.

[3] Faeder JR, Blinov ML, Hlavacek WS (2009) Rule-based modeling of biochemical systems with BioNetGen. *Methods Mol. Biol.* **500**:113–167.

[4] Gillespie DT (1977) Exact stochastic simulations of coupled chemical reactions. *J. Phys. Chem.* **81**:2340–2361.

5 Supplementary figures

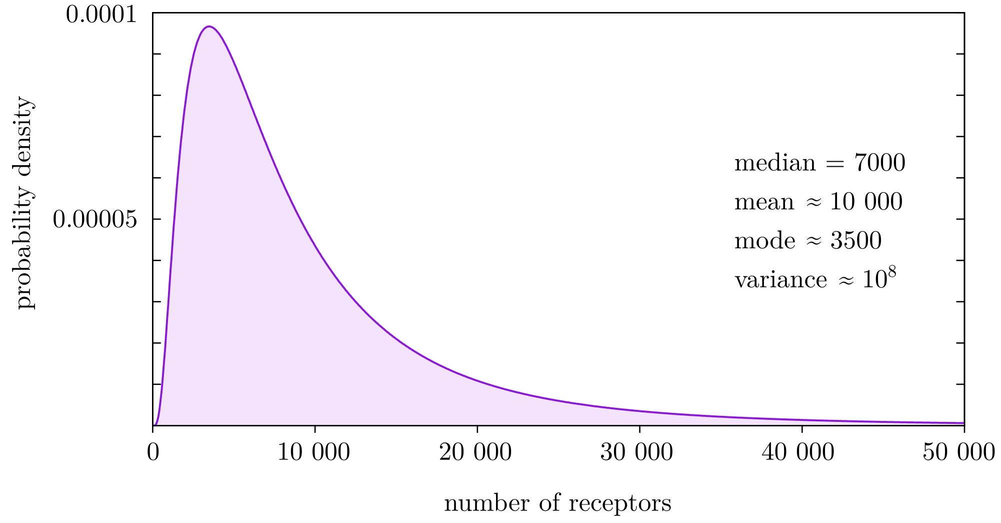

Supplementary Figure S1. The log-normal distribution of the number of TNFR1 receptors.

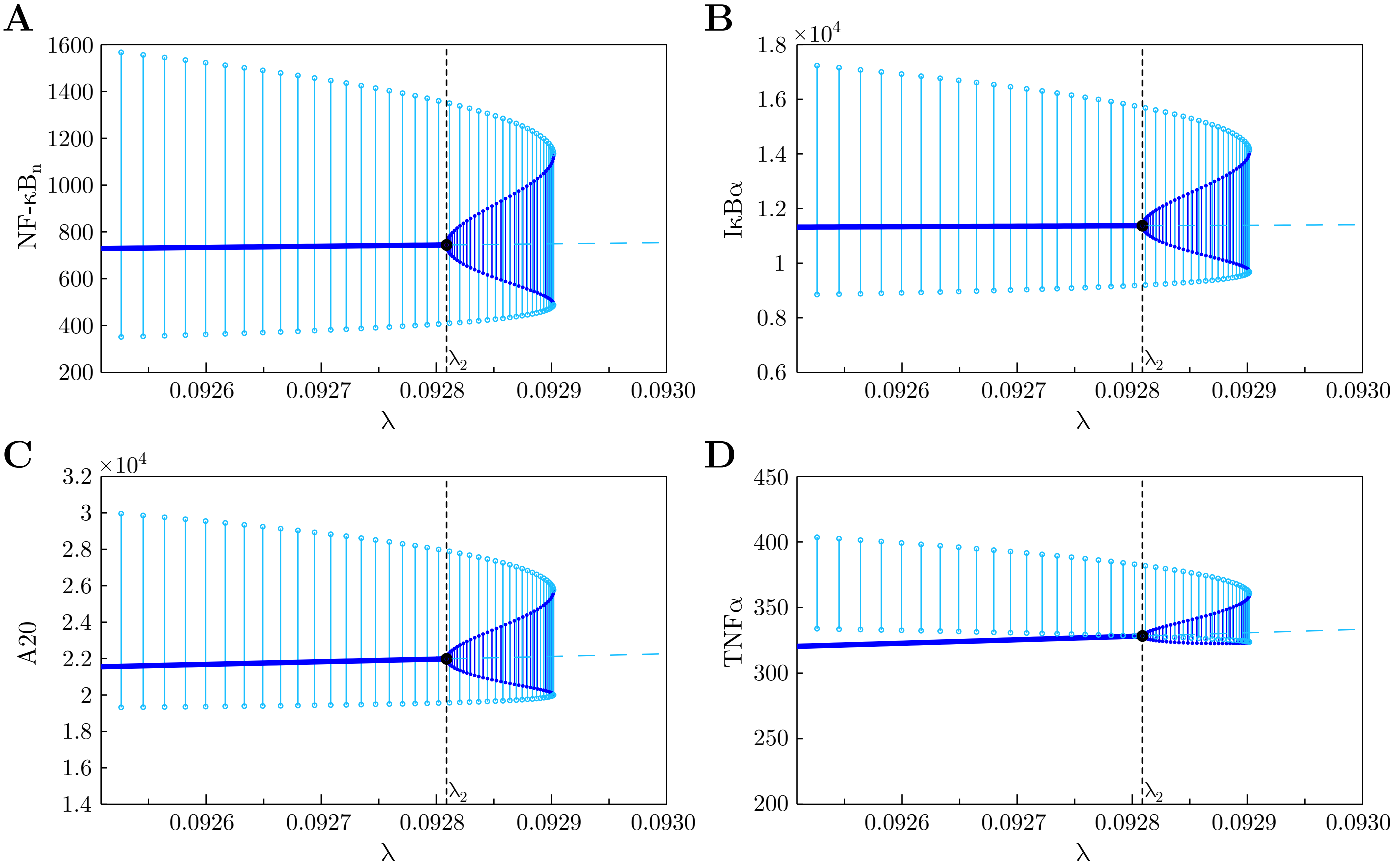

Supplementary Figure S2. Bifurcation diagrams: close-up of the Hopf bifurcation point in λ_2.
Stable steady state – thick dark blue line; unstable steady state – dashed light blue line;
stable limit cycle – filled dots connected by vertical dark blue lines;
unstable limit cycle – small circles connected by vertical light blue lines.

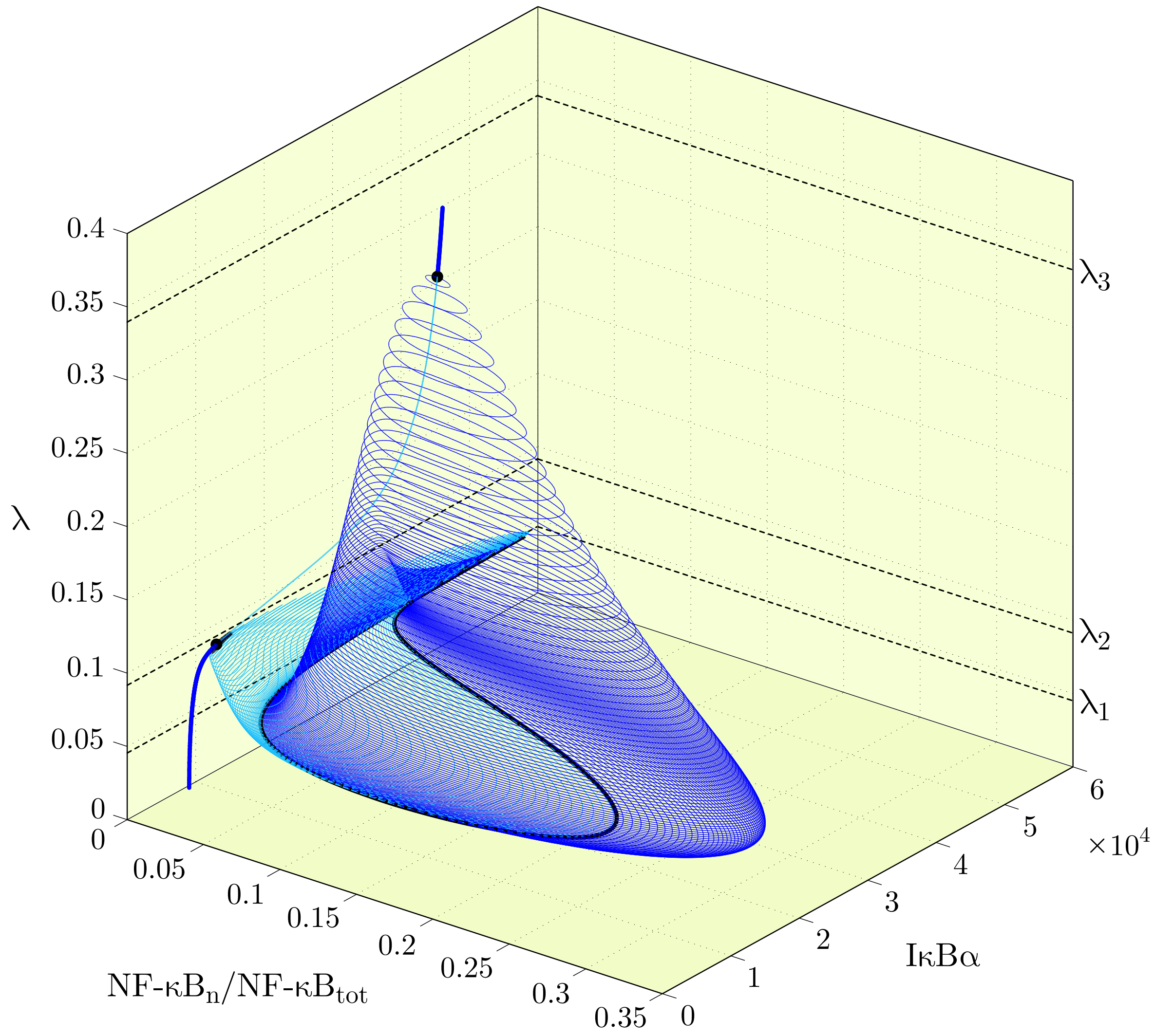

Supplementary Figure S3. Bifurcation diagram for wild-type cells. Stable recurrent states – dark blue, unstable recurrent states – light blue. Cyclic fold at λ_1 and bifurcation points at λ_2, λ_3 – black.

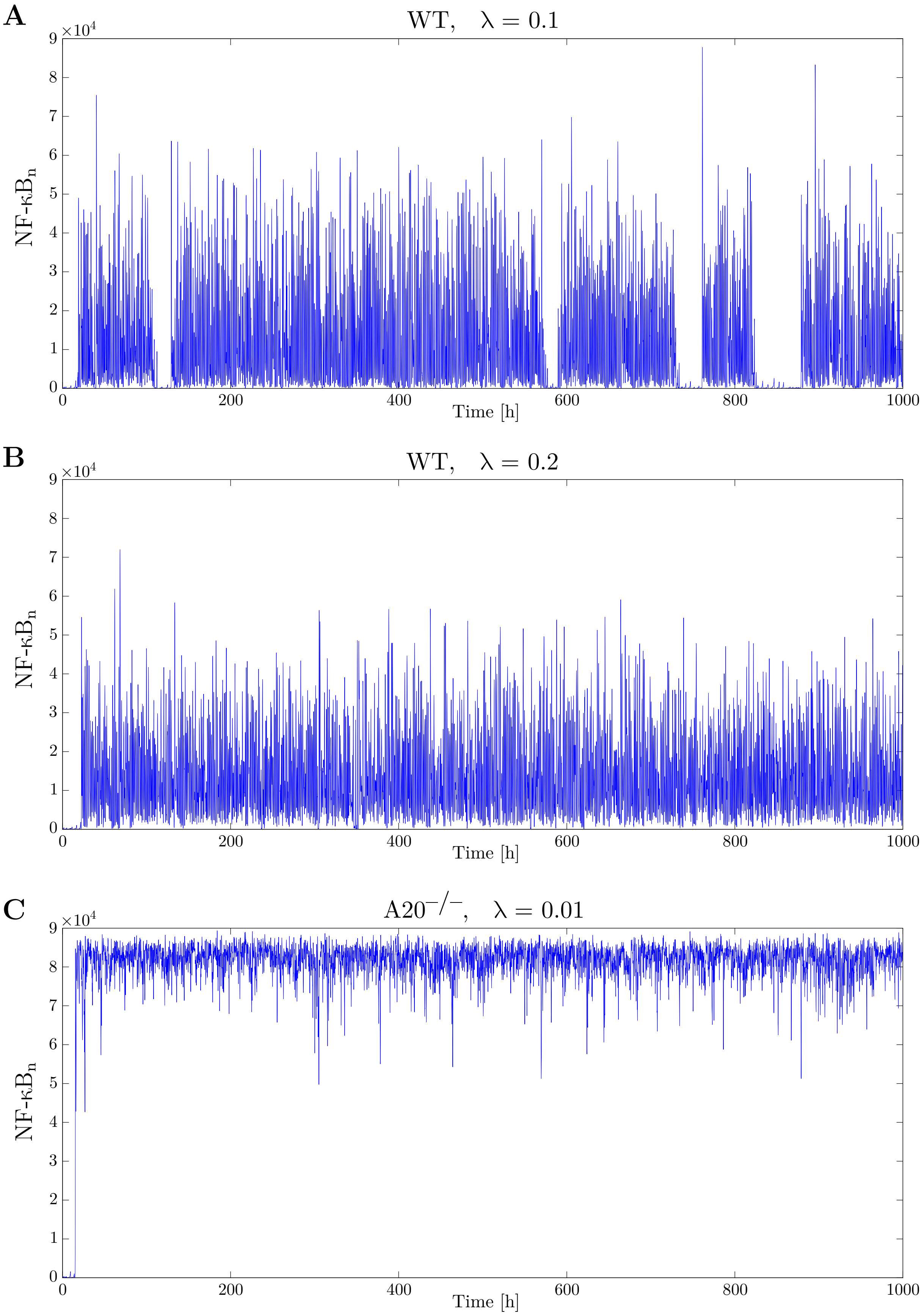

Supplementary Figure S4. Long run stochastic trajectories for wild type (WT) cells for (A) $\lambda = 0.1$, (B) $\lambda = 0.2$, and (C) for A20-deficient (A20$^{-/-}$) cells for $\lambda = 0.01$.
